# Ecological opportunity and adaptive radiations reveal eco-evolutionary perspectives on community structure in competitive communities

**DOI:** 10.1038/s41598-021-98842-8

**Published:** 2021-10-01

**Authors:** Mikael Pontarp

**Affiliations:** Department of Biology, Lund University Biology Building, Sölvegatan 35, 223 62 Lund, Sweden

**Keywords:** Evolution, Evolutionary theory, Phylogenetics, Ecology, Community ecology, Ecological modelling, Evolutionary ecology, Macroecology

## Abstract

It is well known that ecological and evolutionary processes act in concert while shaping biological communities. Diversification can, for example, arise through ecological opportunity and adaptive radiations and competition play an essential role in such diversification. Eco-evolutionary components of competition are thus important for our understanding of community assembly. Such understanding in turn facilitates interpretation of trait- and phylogenetic community patterns in the light of the processes that shape them. Here, I investigate the link between competition, diversification, and trait- and phylogenetic- community patterns using a trait-based model of adaptive radiations. I evaluate the paradigm that competition is an ecological process that drives large trait- and phylogenetic community distances through limiting similarity. Contrary to the common view, I identify low or in some cases counterintuitive relationships between competition and mean phylogenetic distances due to diversification late in evolutionary time and peripheral parts of niche space when competition is weak. Community patterns as a function of competition also change as diversification progresses as the relationship between competition and trait similarity among species can flip from positive to negative with time. The results thus provide novel perspectives on community assembly and emphasize the importance of acknowledging eco-evolutionary processes when interpreting community data.

## Introduction

Overlapping ecological and evolutionary time scales and the importance of eco-evolutionary processes for the assembly of local communities are established^1–3^. Studies show that the diversity of various organisms has arisen through adaptive radiations^4–6^ facilitated by ecological opportunity, i.e. the appearance of empty ecological niches^4,7^. Others emphasize the importance of competition as a driver of such eco-evolutionary diversification. Character displacement due to competition^8^ has, for example, been described in Darwin’s finches^9^. Ecological opportunity, competition for such opportunity, and the diversification process thus affect both phylogenetic and trait patterns in emerging communities. Furthermore, theoretical studies that aim to improve our understanding, as well as our ability to estimate various assembly processes, are receiving increasing attention^10–14^. Several of these studies also identify competition as a major component of community assembly. Combined with established eco-evolutionary theory on frequency-dependent diversification^15,16^ competition thus qualifies as one of the most important determinants of community phylogenetic- and trait-structure.

Understanding how competition may affect eco-evolutionary assembly and community patterns is of importance for understanding community assembly in general^17,18^ but also for inference of processes from community patterns^19–21^. Traditionally, habitat filtering^22^ and competition-driven limiting similarity^23^ have been viewed as dominating assembly processes leading to high and low species similarity, respectively. Such ideas underpin methods that quantify community phenotypic and phylogenetic structure to infer, for example, competition^24^. The focus on relatively simple phenotypic and phylogenetic patterns and the dichotomy of habitat filtering and limiting similarity as two opposing ecological time-scale assembly processes has, however, been criticized^12,25^. Abiotic environmental conditions and depletable resources can be highly correlated and multiple processes act and interact on different spatiotemporal scales^18,19^. Different processes can also give similar patterns^17^ which is problematic given a focus on few processes (e.g. competition) and simple patterns (e.g. community clustering), especially if eco-evolutionary processes are ignored^19,25^. Calls for inference methods that consider evolutionary contingencies have thus been expressed^26,27^. However, for such methods to be effective, clear expectations of how competition affects phylogenies and trait distributions on eco-evolutionary scales, are needed.

**Figure 1:**
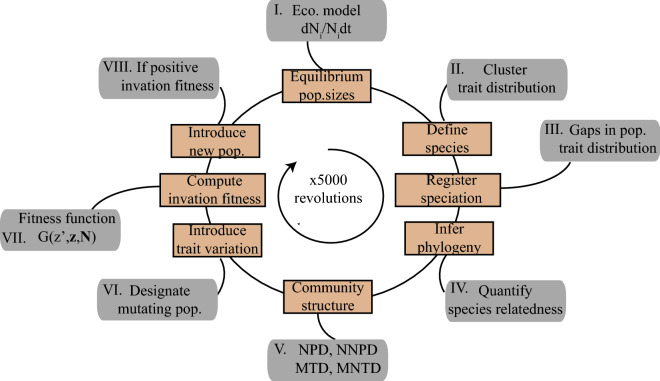
Major components of the eco-evolutionary model implementation and analyses. Each evolutionary time step in the model includes eight components that were conducted in sequence 5000 times (denoted as revolutions in the circular illustration). Light brown boxes denote the purpose of each analysis component and gray boxes illustrate the means or tools for doing so. A trait-based ecological model is used to compute equilibrium population sizes in a given community (**I**). Species are defined according to their traits (**II**), speciation events are registered (**III**) and phylogenic relationships are quantified (**IV**). Community structure (NPD, NNPD, MTD, and MNTD) is quantified (**V**). Trait variation is introduced through mutations (**VI**), invasion fitness of mutants is computed (**VII**) and mutants that have positive invasion fitness are introduced to the community (**VIII**). The next revolution/ evolutionary time step is then started as ecological community equilibrium is re-computed (**I**). Figure produced in Adobe Illustrator CS6 Version 16.0.0.

**Figure 2:**
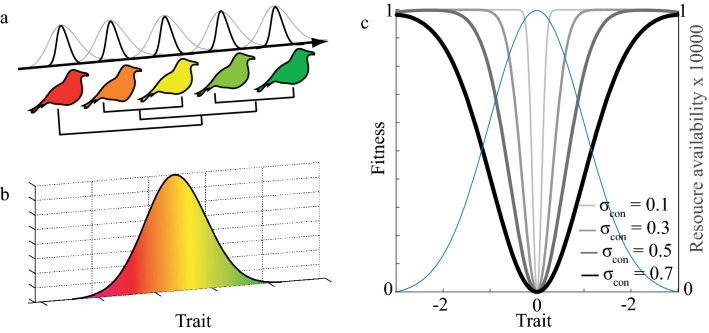
Model﻿ illustration (**a**, **b**) and fitness landscape of a mutant and/ or invading morph at the first branching point of the adaptive radiation (**c**) given different niche widths (σ_con_). A local competitive community (**a**) with some trait (e.g. granivorous birds with some beak size) consumes and competes for resources locally (e.g. habitat or island). Resources are modeled as an implicit continuous resource distribution (**b**) in the same trait dimension as the resource utilization trait here illustrated by color. The fitness of a focal competitor is a function of its trait-matching to its resources, the traits of its competitors, and their niche widths (black and gray niche kernels). If the niche width of the competitors is wide (grey niche kernels) competition strength will be higher in the system than if the niche widths are narrow (black niche kernels). Panel c illustrates resource (blue line) dependent mutant and/ or invader fitness (first y-axis) as a function of trait value (x-axis) with a resident population with trait value = 0 at ecological equilibrium. The seeded monomorphic population (trait value = 0) thus sits on a fitness minimum (branching point) and at equilibrium abundance, the curvature of the fitness landscape and thus the strength of disruptive selection is dependent on niche width (σ_con_) (**c**). The blue line in c denotes resource distribution (second y-axis) as a function of organismal trait (x-axis). Model parameters that were kept constant to compute the fitness landscapes were: *K*_*0*_ = 10,000; *σ*_*K*_ = 1; *r* = 1; *µ* = 0.01; σ_µ_ = 0.02. Figure produced in Adobe Illustrator CS6 Version 16.0.0 and MATLAB version R2019a.

This study aims to investigate the role of eco-evolutionary community assembly processes for the structure of competitive communities. More specifically, with basic assumptions associated with competition and limiting similarity^23^ in mind and similar to Pontarp and Petchey^28^ I simulate the diversification of a local community (Fig. 1). I study the diversification process and emergent community patterns throughout macro-evolutionary history as well as the structure of diversified communities by quantifying mean phylogenetic distance (MPD) and nearest neighbor phylogenetic distance (NNPD). Similarly, I study community trait distributions and trait differences among neighboring species in niche space by quantifying mean trait distance (MTD) and mean nearest trait distance (MNTD). I explicitly connect diversification processes with the mentioned patterns as they emerge and I frame my findings as theoretical expectations on which interpretation of community patterns can rely. To this end, I use a trait-based simulation model of local adaptive radiations to study how trait- and phylogenetic patterns emerge as a consequence of competition-driven eco-evolutionary dynamics (Fig. 1). Based on available adaptive dynamics theory^16^ I focus on eco-evolutionary processes and I model adaptive radiations under the assumption of ecological opportunity^7,29–31^. Following trait-based approaches e.g.^32–34^ I assume that resources are distributed along some generally defined trait dimension and populations are defined by some resource utilization trait in the same trait dimension as the resource (Fig. 2a, b). The niche of the modeled organism thus has two components, the resource that is available in trait space and the organismal trait that dictates resource utilization through the matching between the resource distribution and the resource utilization trait. Utilization, in turn, affects abundance, competition with others, and the evolutionary processes described below. Although generally formulated in the model, an example of the trait dimension can be body size, a trait that has been shown to reflect both resource consumption and competition^35–41^. Furthermore, by assuming that consumer-resource and consumer-consumer trait matching dictates resource utilization and competition respectively, consumer-resource matching will be selected for and consumer-consumer trait matching will be selected against. It follows that the fitness landscape in trait space is determined by the distribution of species and their abundances as well as the resource distribution^25,42,43^. A population in trait space where many similar and abundant populations already exist or where resources are low thus tends to have a low abundance. Similarly, a mutant in trait space where many similar and abundant populations already exist or where resources are low thus tends to have low invasion fitness, here defined as the initial population growth when rare. Contrary, mutants in trait space where resources are available and competition is low have high invasion fitness. Mutant fitness underpins evolutionary dynamics including adaptation and diversification and I utilize these properties of the model to simulate adaptive radiations using three main components of the simulation algorithm. First I implement an ecological model and I compute equilibrium population abundances given resource availability and trait-based inter- and intra-specific competition for those resources (Fig. 1I). Second, I introduce mutations in the ecological traits of the extant populations and I compute mutant invasion fitness (Fig. 1, VII–VIII). Third, I introduce mutants that have positive invasion fitness to the community and I re-compute equilibrium population abundances (Fig. 1, VIII). These major steps ultimately constitute one evolutionary time step and for each step, I designate each population to a species based on distinct trait clusters as they emerge from one common ancestor (Fig. 1, II–IV). By iterating over evolutionary steps, trait distributions and phylogenetic patterns emerge as the adaptive radiation is simulated. By registering mean species traits and the time and origin of speciation events I analyze phylogenetic and phenotypic community structure (MPD, NNPD, MTD, MNTD) over eco-evolutionary time scales (Fig. 1, V). The approach thus provides an explicit and link between adaptive dynamics theory, trait data and phylogenetic patterns. The approach thus also linking called for knowledge on diversification processes and observable community patterns. See the methods section for details on the ecological model, the fitness analysis, the simulation algorithm, and community structure analyses.

## Results

**Figure 3:**
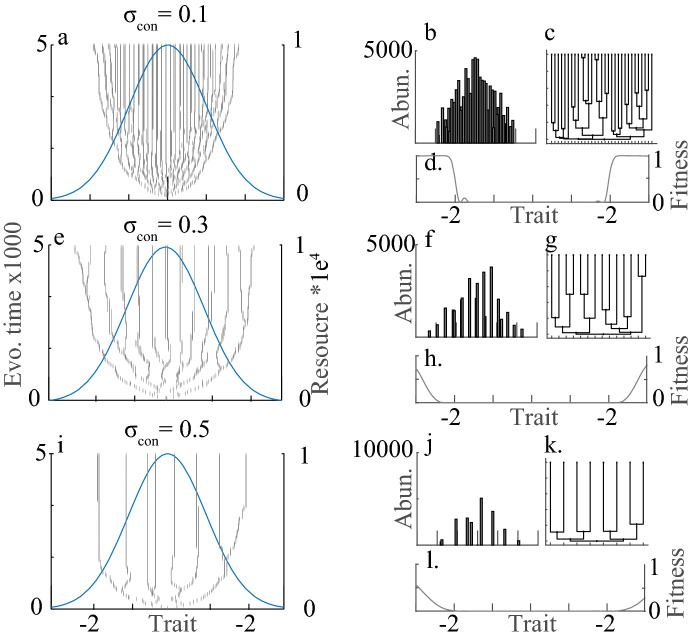
Adaptive radiations (**a**, **e** and **i**), trait distributions at evolutionary time 5000 (**b**, **f** and **j**), phylogeny (**c**, **g** and **k**) and fitness landscape at time 5000 (**d**, **h** and **l**) for simulations with species niche widths (σ_con_) being set to 0.1 (**a**–**d**), 0.3 (**e**–**h**) and 0.5 (**i**–**l**). Each simulation was seeded with one monomorphic competitive population with a trait value equal to zero. Simulations were run for 5000 evolutionary time steps where each time step involves potential invasions of mutants and equilibrium population size computation. Community dynamics on both ecological (population dynamics) and evolutionary (macro-evolutionary dynamics) time scales are dictated by the consumer-resource and consumer-consumer trait matching as well as niche width and resource availability. Blue lines in a,e and i shows the resource distribution (second y-axis). Model parameters that were kept constant for the simulations were: *K*_*0*_ = 10,000; *σ*_*K*_ = 1; *r* = 1; *µ* = 0.01; σ_µ_ = 0.02. Phylogenetic trees were constructed using data on the distance to the nearest common ancestor between the species at the 5000th simulation time step and the MATLAB function *seqlinkage*. Figure produced in MATLAB version R2019a.

**Figure 4:**
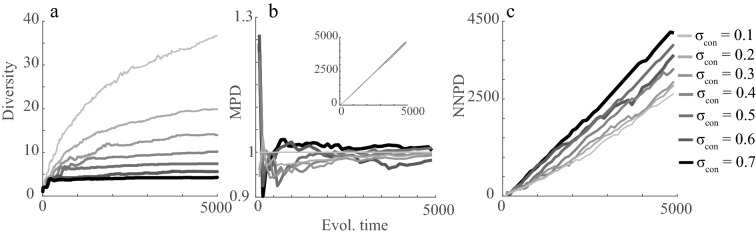
Diversity (**a**), mean phylogenetic trait distance (MPD) (**b**) and nearest neighbor phylogenetic distance (NNPD) (**c**) as a function of evolutionary time and niche widths (σ_con_). MPD in (**b**). is scaled using σ_con_ = 0.1 as reference and the y-axis thus shows the change factor of MPD at different σ_con_ values compared to the σ_con_ = 0.1 baseline. Insert in (**b**). show non-scaled MPD values as a function of evolutionary time and σ_con_, indicating a low variation among σ_con_ –scenarios and a monotonic increase in MPD with time. All results are shown as means over 20 simulation replicates (see variation across replicates in Fig. S1). Model parameters that were kept constant for the simulations were: *K*_*0*_ = 10,000; *σ*_*K*_ = 1; *r* = 1; *µ* = 0.01; σ_µ_ = 0.02. Figure produced in MATLAB version R2019a.

Results show a negative relationship between niche width, diversification, and community richness (Figs. 3, 4a) with low variation across replicates (Fig. S1). This is expected and aligns with results on diversification studied in other contexts^15,16,23,28,44^. Understanding the underpinning of such diversification is imperative for an understanding of trait- and phylogenetic community patterns. Analysis of the fitness landscape (Eqs. 5–8) provides part of the explanation. When niche width is narrow and when few species occupy the community (e.g. at the start of the simulations with one seeded population) the curvature of the fitness landscape at a branching point is large compared to when niche width is broad (Fig. 2c). The disruptive selection at branching points in trait space is thus also strong which may result in relatively fast branching (see also^28^). As evolutionary time progresses, diversification decreases, and a steady-state in terms of richness is reached (Figs. 3, 4). At this point, when niche space is filled, no mutants have positive invasion fitness and the diversification process ends, is reached at lower richness when niche widths are wide compared to when narrow niche widths are modeled (Figs. 3, 4).

As evolutionary time and diversification progresses the phylogenetic-based metrics (MPD and NNPD) increase continuously as the communities are assembled (Fig. 4b, c). Such an increase is expected as both MPD and NNPD are based on the evolutionary time since the species first occurred. As the community reaches a steady-state, species get older, and the phylogenetic distance between species increase. Interestingly, results do however not show a large difference in MPD as a function of niche width and the relationship can vary with evolutionary history (Fig. 4b). In contrast, a positive relationship between NNPD and niche width is shown, and this relationship is enhanced as diversification progresses (Fig. 4c).

**Figure 5:**
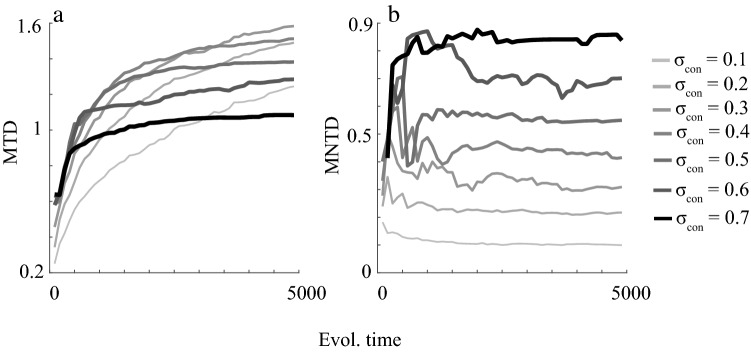
Mean trait distance (MTD) (**a**) and mean nearest trait distance (MNTD) (**b**) as a function of evolutionary time and niche widths (σ_con_). All results are shown as means over 20 simulation replicates. Model parameters that were kept constant for the simulations were: *K*_*0*_ = 10,000; *σ*_*K*_ = 1; *r* = 1; *µ* = 0.01; σ_µ_ = 0.02. Figure produced in MATLAB version R2019a.

Furthermore, results show a positive relationship between MTD and niche width early in the radiations but the relationship can flip from positive to negative with evolutionary time (Fig. 5a). The explanation of such a flip is based on the niche packing process. As niche packing occurs MNTD increases at first, followed by a slight decrease, and then it stabilizes (Fig. 5b). Communities with wide niche widths contain few species that tend to be evenly distributed and far apart in trait space (Fig. 3i–k) compared to narrow niche width scenarios leading to a positive relationship between MNTD and niche width (Fig. 5b). As a result, narrow niche width scenarios have low MTD initially as species that are close in trait space can co-exist (Fig. 5b). However, as species diversify and more species occupy peripheral parts of trait space MTD tends to increase and pass wide niche width scenarios leading to the flip in the competition-MTD relationship (Fig. 5b). All results are robust to the simulation implementation for updating evolutionary time, population growth rate (*r*) ranging from 0.75–1.25, variation in evolutionary potential modeled as mutational variance (σ_µ_) ranging from 0.01–0.04, and model design in terms of the shape of the resource distribution kernel (Figs. S2, S3, S4, S5).

## Discussion

The importance of eco-evolutionary processes for community assembly is established and competition, which is directly related to overlapping niche space and thus also niche width, has proven to be essential for diversification in eco-evolutionary models. When niche width is narrower than the variation in resources (i.e. σ_con_ < σ_K_ in my model) co-existence of two or more species is possible and local evolutionary branching is facilitated^15,16^. Such theory also shows how communities can be structured differently through invasions from an external species pool compared to gradual local adaptation and speciation^45^. Still, many current inference techniques ignore evolutionary processes and instead assume an available species pool from which a community is assembled on ecological time scales only^19,25,26,46^.

In this study, I abandon the assumption of an available species pool and instead embrace evidence for the importance of eco-evolutionary processes^1–3^. In a way, I focus on the extreme contrast to the “species pool assumption” by modeling the environment as a depletable resource, simulating adaptive radiations and quantify the metrics on which several community patterns (e.g. phylogenetic clustering) rely^24^. The model thus provides novel results as it highlights different ecological and evolutionary processes, all of which can be linked to competition, that affect community patterns in unexpected ways. As expected, the degree of competition in a community dictates species coexistence, diversification through adaptive radiations, and species richness (Figs. 3, 4). The competition also affects the strength of disruptive selection at branching points (Fig. 2c), potentially affecting the speed of diversification and thus phylogenetic community structure. The results thus reveal two main macro-evolutionary effects that have implications for emergent trait- and phylogenetic- patterns. First, diversification reaches a steady-state early in evolutionary time in wide niche width scenarios compared to when niche widths are narrow, ultimately affecting the time that phylogenetic differences accumulate. Second, diversification in peripheral parts of niche space tends to occur when niche widths are narrow, affecting the mean trait- and phylogenetic- differences in the system. Both effects are rarely recogniced in the literature but provide insight into some of the patterns presented above that go against the current paradigm of a negative relationship between competition and trait- and phylogenetic-similarity.

The model suggests a small difference in MPD as a function of niche width (competition) although it is built on assumptions associated with classical niche theory and limiting similarity. The result thus contradicts the common idea that species should be less similar if they compete a lot. A visual inspection of the phylogenies (Fig. 3), the γ-statistics of the phylogeny (Fig. S6), and the distribution of branch lengths and phylogenetic distances (Figs. S7, S8) explain the result. When competition is low, similar species can coexist which facilitates diversification and high richness as well as later branching events in peripheral parts of niche space. Many closely related species thus co-exist across a large span of niche space but the change in the distribution of phylogenetic distances across the full phylogeny remains low across niche width scenarios (Fig. S8). A similar explanation can be used for the results showing that the relationship between competition and trait similarity among species can flip from positive to negative with evolutionary time (Fig. 5a). A positive relationship between MTD and niche width early in the radiations align with the paradigm that competition tends to give high MTD. However, communities with wide niche widths reach diversity equilibrium and an asymptotic MTD early. Narrow niche width scenarios, on the other hand, have low MTD initially but as species diversify for longer into peripheral parts of trait space MTD increases.

The effect of time until a steady-state and diversification in peripheral parts of niche space also open up for alternative explanations for seemingly expected results. For example, a positive relationship between NNPD and niche width (Fig. 4c) aligns with the common paradigm. Previous studies also emphasize that signals in NNPD better reflect patterns that are due to competition than other metrics^21^. My results corroborate such results combined with an emphasis on a macro-evolutionary dimension to the explanation. NNPD is increasing as the diversification progresses irrespective of the strength of competition but NNPD accumulates faster when equilibrium is reached and no more closely related species emerge. As shown by the accumulation of diversity and the γ-statistics of the phylogeny, a steady state is reached faster and NNPD will accumulate faster in high niche width cases compared to when niche widths are narrow (Fig. 4c).

The results discussed above have implications for methods that infer competition from trait- or phylogenetic- overdispersion (associated with high MPD, NNPD, MTD, and MNTD) using species pool and null model approaches^47^. My results support the idea that competition can structure communities to be dissimilar but alternative explanations are also plausible. Low species similarities across a community can, for example, be the result of slow diversification or old communities that reached a steady-state a long time ago. The assumption that MPD should be relatively high in communities associated with competing species may not always be correct as competition also affects diversification in peripheral parts of niche space which ultimately affects mean phylogenetic distances. High NNPD may be due to high competition and fast niche filling followed by static communities (e.g. in old or non-radiating communities). On the other hand, high MTD values can be due to both high competition leading to niche spacing or due to low competition facilitating radiation of many species into a wide range of niche space. Such alternatives explanations are testable but, as in any model, simplification and specific assumptions need to be considered while testing theory.

I focus explicitly on adaptive radiations^7,29^ and for generality, I model asexual organisms and I assume a constant environment and resource availability. I assume a one-dimensional trait space, I make explicit assumptions about competition and resource utilization through trait matching and I model one habitat only. Such generality facilitates a novel understanding of the fundamental link between established eco-evolutionary processes and community structure. With this said, it may seem unrealistic to compare communities that contain species with non-evolving narrow or broad niche widths, respectively. Especially as it has been shown that niche filling can occur either through specialization which facilitates diversification or through the evolution of generalist strategies^48,49^. Such effects need to be considered in the wider context of community assembly and process inference. For example, the evolution of generalist strategies may reduce diversification and potentially increase phenotypic and phylogenetic distances in the community. Nevertheless, given the scope of this study, I do not allow for the evolution of generalist strategies at the expense of diversification. Furthermore, it can be argued that community assembly through adaptive radiations is rare. Instead, both ecological (e.g. colonization) and eco-evolutionary (e.g. local adaptation and adaptive radiation) processes are likely to be active at the same time^50,51^ and spatial contingencies have been shown to affect diversification and thus community patterns^25,42,52^. Previous studies focus on the way invasions into communities can affect community assembly in general^10^ and trait metrics like MTD^53^ in particular. The true complexity of community dynamics and structure is likely a mix of many processes including the ones mentioned above and the relative importance is likely context-dependent. In this study, I isolate basic eco-evolutionary processes, focus on competition and adaptive radiations, and exclude some of the complications listed above. Phylogenetic and phenotypic community patterns are modeled as emergent properties given minimal but fundamental assumptions. I avoid processes that are out of scope for this study that may complicate or mask results. This leads me to conclude that ubiquitous ecological processes like competition can affect community patterns in a range of ways when studied across eco-evolutionary time scales but such effects are not ubiquitous or exclusive. A combination of ecological time scale effects e.g.^53^ and evolutionary effects are likely to occur in concert^21,27,50,51^ and we need to study processes both in combination and in isolation. The results presented here certainly suggest caution in the way community patterns are interpreted, especially if eco-evolutionary processes are suspected to be active. It will be intriguing to follow how future inference methods may develop to tackle the challenge of using functional resource trait- and phylogenetic patterns to infer interacting ecological and evolutionary assembly processes^11,12,14,25,54^.

## Methods

### Ecological model

The model builds on the generalized Lotka–Volterra (GLV) model for *n* competing populations e.g.^55,56^. The ecological dynamics, in per capita form, of the populations are described as:

1 $$\frac{{dN_{i} }}{{N_{i} dt}} = r_{i} + \mathop \sum \limits_{j = 1}^{n} \frac{{ - r_{i} \alpha_{ij} N_{j} }}{{K_{i} }}$$

for *i* = 1 to *n* and where *N*_*i*_ denotes population size, *r*_*i*_ is the intrinsic growth rate and *K*_*i*_ denotes the carrying capacity of population *i*. The parameter $\alpha_{ij}$ denotes the ecological interaction, in this case, competition, between populations’ *i* and *j*. Similar to other trait-based models ^15,34,57^, I expand on the model by introducing trait-based interactions and I describe the competitive community with a dynamic vector **N**, representing population abundance, and a static (on ecological time scale) vector **z**, representing the population traits. More specifically, I reformulate carrying capacity (*K*_*i*_) and the interactions ($\alpha_{ij}$) as trait dependent functions:

2 $$K\left( {z,z_{opt} } \right) = K_{0} e^{{ - { }\frac{{\left( {z_{opt} - z} \right)^{2} }}{{2\sigma_{K}^{2} }}}}$$

and

3 $$\alpha \left( {z,z_{j} } \right) = e^{{ - { }\frac{{\left( {z - z_{j} } \right)^{2} }}{{2\sigma_{con}^{2} }}}}$$

The full trait-based ecological model thus expands to:

4 $$\frac{{dN_{i} }}{{N_{i} dt}} = r_{i} + \mathop \sum \limits_{j = 1}^{n} \frac{{ - r_{i} \alpha \left( {z_{i} ,z_{j} } \right)N_{j} }}{{K\left( {z_{i} ,z_{opt} } \right)}}$$

where *K*(z, *z*_*opt*_) represents the carrying capacity for a monomorphic population of individuals with trait value *z* in the habitat characterized by a resource distribution with its peak resource availability at the point *z*_*opt*_. For simplicity in the evolutionary analyses of the model (see below) I assume *z*_*opt*_ to be equal to zero. *K*_0_ denotes the maximal carrying capacity (at *z* = *z*_*opt*_) and it follows from Eq. (2) that the resource availability declines symmetrically as *z* deviates from *z*_*opt*_ according to the width of the resource distribution (*σ*_*K*_) (Fig. 2). Equation (3) models the interaction coefficient, *α*(*z,z*_*j*_), between the focal population (defined by its trait *z*_*i*_) and its competitors (defined by their traits *z*_*j*_). Here, I standardize the competition coefficients so that, for a focal population *i*, α_ii_ = 1 and 0 < α_ij_ < 1 (*z*_*i*_ ≠ *z*_*j*_). *σ*_con_ is an essential parameter for this particular study as it determines the degree of competition between individuals given certain utilization traits.

### Evolutionary analysis

I start by investigating the evolutionary dynamics of a single population in a habitat with biotic conditions defined by the resource distribution (Eq. 2). This has relevance for the interpretation of results as well as the initiation of the adaptive radiation simulations (see simulation details below). I use the adaptive dynamics framework^16,28,58^ which assumes the introduction of small mutations in trait space and that the focal population at equilibrium. Here, the resource utilization trait (*z*) is under selection with the potential to evolve and our intuitive expectation should be that selection drives adaptation to the optimal resource trait value *z*_*opt*_. Mathematically the fitness of any given mutant trait (z′) for any given condition defined by **N** (community richness and abundance) and **z** (trait distribution) is described as:

5 $$G\left( {z^{{\prime }} ,{\mathbf{z}},{\mathbf{N}}} \right) = r + \mathop \sum \limits_{j = 1}^{n} \frac{{ - r\alpha \left( {z^{{\prime }} ,z_{j} } \right)N_{j} }}{{K\left( {z^{{\prime }} ,z_{opt} } \right)}}$$

Note that the notation in Eq. (5) is general for the fitness of a mutant competing with any number of species in a community setting. In the single population case, **z** and **N** reduce to scalars. The slope of this fitness function (Eq. 5) dictates the direction and speed of evolution in *z*. The fitness gradient of a population seeded into my simulation model analysis is thus formulated as:

6 $$\left. {\frac{\partial G}{{\partial z^{{\prime }} }}} \right|_{{\left( {z^{{\prime }} = z, N = N^{*} \left( z \right)} \right)}} = - \frac{r}{{\sigma_{K}^{2} }}z$$

where N* denotes equilibrium population size. It follows that the fitness gradient is positive for *z* < 0, negative for *z* > 0 and zero at *z* = 0 = *z*_*opt*_.

Differentiating Eq. (6) with respect to *z* gives:

7 $$\frac{d}{dz}\left( {\frac{\partial G}{{\partial z^{{\prime }} }}} \right) = - \frac{r}{{\sigma_{K}^{2} }}$$

and *z* = 0 is thus always a convergent stable evolutionary singular point. A population of individuals with trait *z* away from zero will always evolve towards *z* = 0. What happens to the population at trait value *z* = 0 can be analyzed by the second-order partial derivative of the fitness function with respect to *z′.* For this model, the derivative formulates as:

8 $$\left. {\frac{{\partial^{2} G}}{{\partial z^{{{\prime }2}} }}} \right|_{{\begin{array}{*{20}c} {z^{{\prime }} = 0} \\ {z = 0} \\ {N = N^{*} \left( 0 \right)} \\ \end{array} }} = r\left( {\frac{1}{{\sigma_{con}^{2} }} - \frac{1}{{\sigma_{K}^{2} }}} \right)$$

This tells us that selection is disruptive at *z* = 0 and evolutionary branching (trait divergence and potential speciation) can occur if mutant populations are allowed to invade and if σ_con_ < σ_*K*_ (Fig. 2c). If σ_con_ > σ_*K*_ the selection is stabilizing and no branching will occur^59^. I build on this information for the initiation of the adaptive radiation simulations.

### Simulation algorithm and parameter values

I use the information from the evolutionary analyses presented above in my initiation of the adaptive radiation simulations. If σ_con_ is larger than or close to the width of the resource distribution σ_K_ (here set as a constant = 1), competition strength will be high even between populations utilizing opposite ends of the local resource distribution. σ_con_ values close to 1 thus denote a scenario of high competition and low diversification potential. As my main interest is to elucidate the effect of competition strength I investigate simulation scenarios denoted by niche widths (σ_con_) ranging from 0.1–0.7 in increments of 0.1. I seed the system for each of the scenarios with one monomorphic population at trait value equal to 0 with abundance equal to 1 and I compute equilibrium population size. Similar to, for example, Ito and Dieckmann^60^ I iterate over 5000 steps of (i) introduce mutations, (ii) compute mutant invasion fitness, (iii) compute mutant and resident mutual invasibility and (iv) add the mutant population to the community or replace the mutating population with the mutant population, and (v) re-computing equilibrium population sizes (see also Fig. 1). More specifically, I compute the equilibrium population sizes by integrating over Eq. (4) until equilibrium or a steady state is reached (integrating from time 0 to 1000 is proven sufficient). Populations mutate according to the product of the population size and mutation probability (*µ*) equal to 0.01. I thus draw a single mutant at each evolutionary time step with probability weighted by population sizes and the mutation probabilities. The mutant trait is modeled as a random value drawn from a normal distribution with a mean equal to the trait of the mutating population and a variance (σ_µ_) equal to 0.02. I compute invasions fitness by solving Eq. (5) numerically and if invasion fitness is positive, I do a mutual invasibility test to see if the resident morph can invade the mutant if at equilibrium. If mutual invasibility exists then I introduce the mutant alongside the resident, otherwise, I replace the resident with the mutant morph. I then re-compute the equilibrium and delete extinct populations. I ran the evolutionary dynamics for 5000 steps, which proved enough to reach a community at, or close to, evolutionary equilibrium (Fig. 3) for the given parameters (see^44,61^ for detailed exploration of evolutionary equilibrium in similar models).

For each evolutionary step described above, I also assigned each population to a species id using a trait-based speciation definition see also^28,42,43^. I define species as populations having common descent and continuous distribution of traits (no gaps in the trait distribution > 3 × σ_µ_). When I detected a gap > 3 × σ_µ_ in the trait distribution within an existing species, I considered it a speciation event (i.e. one species branching into two). Although somewhat arbitrary, this limit of 3 × σ_µ_ makes biological sense as it is large enough to prevent speciation by only a few mutations^28,52^. By registering the time and origin of all speciation events as well as mean species traits and abundance for each evolutionary step I have all the information required to follow the phylogenetic and phenotypic community structure as a function of evolutionary time as it is defined by the simulation algorithm (see community structure analyses below).

Parameters that were kept constant for the simulations were: *K*_*0*_ = 10,000; *σ*_*K*_ = 1; *r* = 1; *µ* = 0.01; σ_µ_ = 0.02. These constants were chosen to produce diverse enough communities to analyze community structure within reasonable computational time. It follows from Eqs. (5–8) that these parameters, except for σ_K_ which should be considered in relation to σ_con_ (Eqs. 2–4) are not expected to affect the results and conclusions as they are not affecting the fitness landscape at equilibrium nor the sequential branching process of the adaptive radiations. However, these parameters may affect evolutionary time and evolvability. More specifically, *r* affects the time it takes for community equilibrium to be reached and should thus be considered for the numerical analysis of the population dynamics. The other constants *K*_*0*_, *µ* and σ_µ_ affect the speed of evolution as these parameters affect the number and size of mutations. These parameters should thus be interpreted in relation to the number of evolutionary time steps run in the simulation model. Additional analyses were conducted to confirm the robustness of results given decisions on the rate of ecological dynamics (*r*), evolvability (σ_µ_) and model design in terms of kernels used for carrying capacity (Figs. S2, S3, S4, S5). Robustness checks were also done to the eco-evolutionary implementation of modeling evolutionary time steps. Although parsimonious this way of modeling evolutionary time does not directly translate into the common time component of phylogenies (e.g. years). An alternative way is to model time explicitly as a function of the total rate of mutation in the system (*w*) according to Δt = − (1/*w*)lnρ, where 0 < ρ $\underline{ < }$ 1 is a uniformly distributed random number^60^. The results were, however, robust to such alternative analyses as the sequence or branching’s do not rely on *r* and σ_µ_ nor on the way time is modeled. Code for the model implementation is available (https://doi.org/10.5281/zenodo.5342486).

### Community structure

I base my analysis on the phylogenetic distance (based on time for speciation) and on the position in trait space among species (based on the mean trait of species). In line with Webb et al.^24^, Webb et al.^62^ and Harmon-Threatt and Ackerly^63^ and similar to Pontarp and Petchey^53^ I compute the community structure, as mean phylogenetic distance (MPD), nearest neighbor phylogenetic distance (NNPD), mean trait distance (MTD) and mean nearest trait distance (MNTD). MPD and MTD calculate the mean phylogenetic and trait distance separating all species in a community while NNPD and MNTD calculate the mean distance, in trait- or phylogenetic space, between the species’ and their nearest neighbor. I quantify these metrics for every 100 evolutionary time steps and given that some of the stochastic components (e.g. introduction of mutants) of the evolutionary simulation algorithm can generate variation between model realizations I run 20 replicates of each assembled competitive consumer community. Furthermore, to corroborate some of the mechanistic explanations of some of the effects seen on the distance metrics presented above I use the γ-statistics^64^, a metric of the temporal distribution of branching events across the phylogenies.

## Supplementary Information

Supplementary Information.
