# Supplementary material for: Ecological opportunity and adaptive radiations reveal eco-evolutionary perspectives on community structure in competitive communities
Source: Sci Rep. 2021 Oct 1;11:19560. doi: 10.1038/s41598-021-98842-8 (PMC8486866; doi:10.1038/s41598-021-98842-8)
Supplement: Supplementary file 1 — Supplementary Information. [file 41598_2021_98842_MOESM1_ESM.docx]

# Ecological opportunity and adaptive radiations reveal eco-evolutionary perspectives on community structure in competitive communities

Mikael Pontarp

Department of Biology, Lund University Biology Building, Sölvegatan 35, 223 62 Lund, Sweden

**Supplementary Information**

**Figure S1**

**
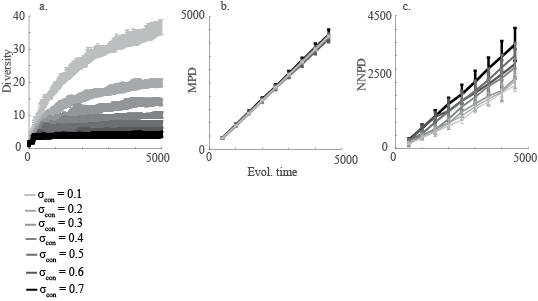
**

Diversity (a), mean phylogenetic trait distance (MPD) (b) and nearest neighbor phylogenetic distance (NNPD) (c) as a function of evolutionary time and niche widths (σ_con_). Error bars denote variation across 20 simulation replicates computed as standard deviations. Model parameters that were kept constant for the simulations were: *K_0_*=10000; *σ_K_* =1; *r* = 1; *µ*=0.01; σ_µ_ =0.02. Figure produced in MATLAB version R2019a.

**Figure S2**

**
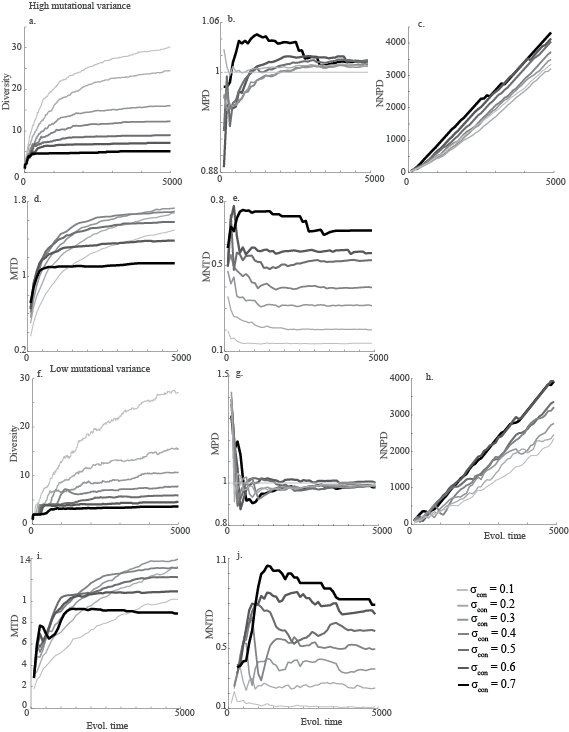
**

Diversity, mean phylogenetic trait distance (MPD) and nearest neighbor phylogenetic distance (NNPD) as a function of evolutionary time and niche widths (σ_con_) for high (σ_µ_ =0.01) (a-e) and low (σ_µ_ =0.04) (f-j) mutational variance. MPD is scaled using σ_con_ = 0.1 as reference and thus show the change factor of MPD at different σ_con_ values compared to the σ_con_ = 0.1 baseline. Model parameters that were kept constant for the simulations were: *K_0_*=10000; *σ_K_* =1; *r* = 1; *µ*=0.01. Figure produced in MATLAB version R2019a.

**Figure S3**

**
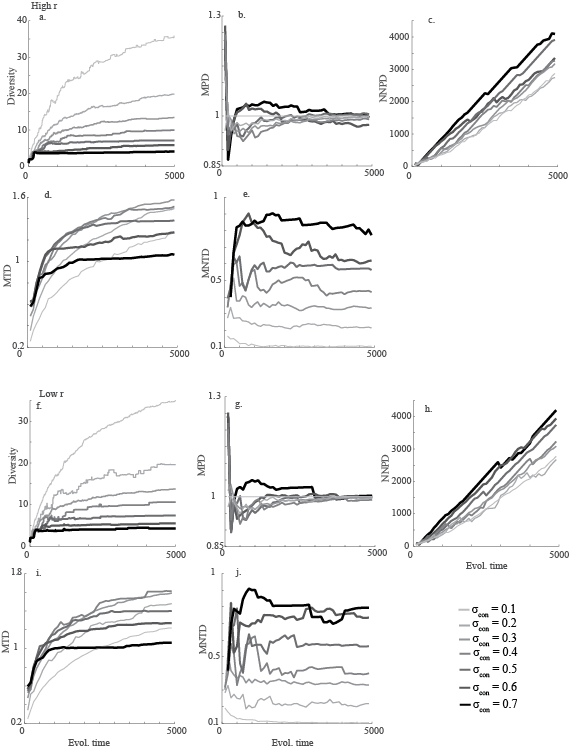
**

Diversity, mean phylogenetic trait distance (MPD) and nearest neighbor phylogenetic distance (NNPD) as a function of evolutionary time and niche widths (σ_con_) for high (*r* =0.01) (a-e) and low (*r* =0.04) (f-j) growth rate. MPD is scaled using σ_con_ = 0.1 as reference and thus show the change factor of MPD at different σ_con_ values compared to the σ_con_ = 0.1 baseline. Model parameters that were kept constant for the simulations were: *K_0_*=10000; *σ_K_* =1; *µ*=0.01; σ_µ_ =0.02. Figure produced in MATLAB version R2019a.

**Figure S4**

Results from an alternative simulation implementation where evolutionary time progresses proportional to mutation rate. Time is updated for each evolutionary simulation step according to -(1/w) ln p, where w is the total mutation rate in the system at a given time and 0<p<1 is a uniform distributed random number (Ito & Dieckmann 2007). Diversity (a), mean phylogenetic trait distance (MPD) (b), nearest neighbor phylogenetic distance (NNPD) (c), mean trait distance (MTD) (d) and mean nearest trait distance (MNTD) (e) as a function of evolutionary time and niche widths (σ_con_). MPD in b. is scaled using σ_con_ = 0.1 as reference and thus show the change factor of MPD at different σ_con_ values compared to the σ_con_ = 0.1 baseline. Model parameters that were kept constant for the simulations were: *K_0_*=10000; *σ_K_* =1; *r* = 1; *µ*=0.01; σ_µ_ =0.02. Figure produced in MATLAB version R2019a.

**Figure S5**


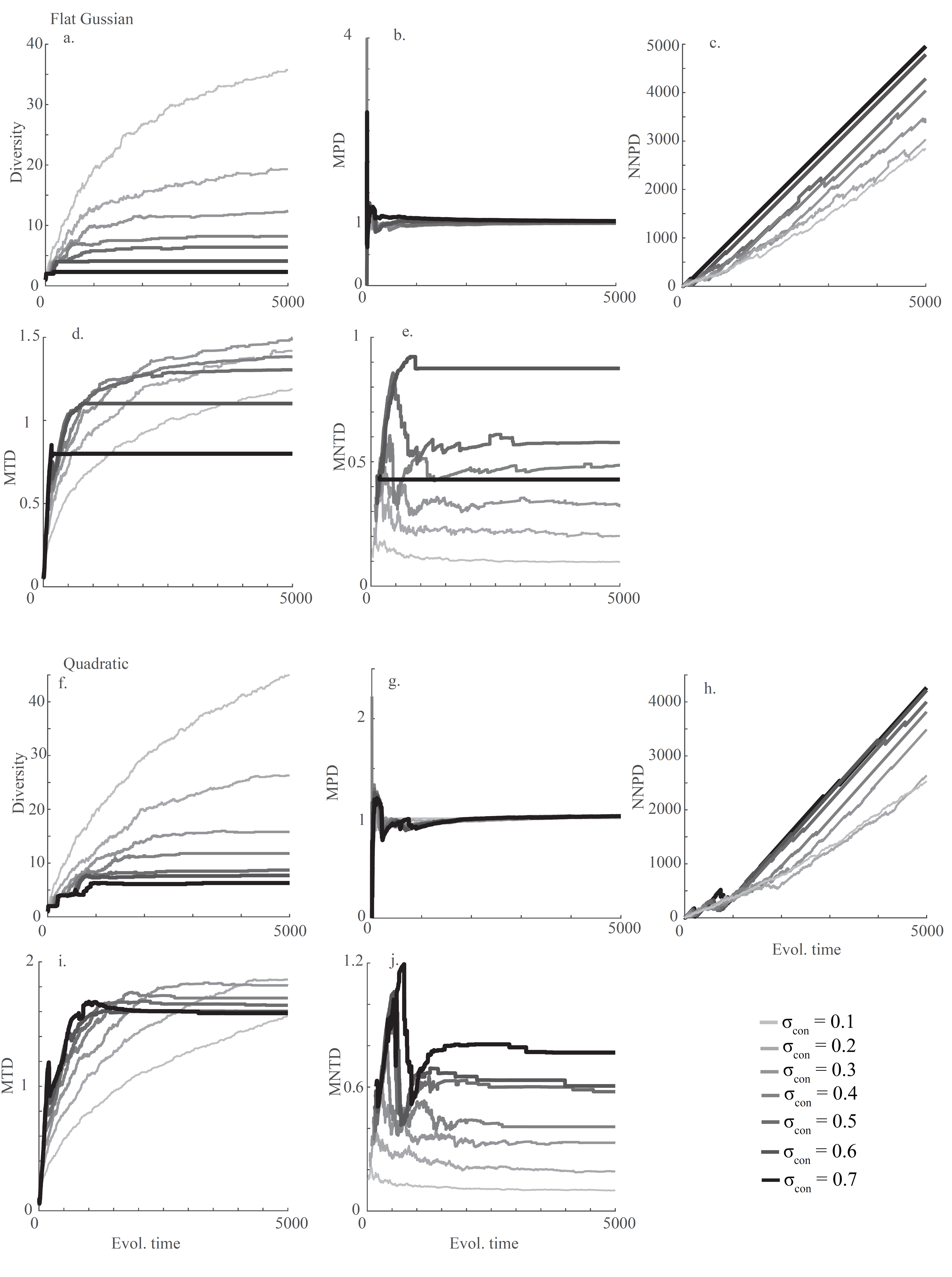


Diversity, mean phylogenetic trait distance (MPD) and nearest neighbor phylogenetic distance (NNPD) as a function of evolutionary time and niche widths (σ_con_) using a flat Gaussian kernel (*K_0_**exp(-((z_opt_-z)^2/2/ σ_K_ ^2)^1.2)) (a-e) and quadratic kernel (*K_0_**(1-(z_opt_-z)^2/ σ_K_ ^2)) (f-j) for carrying capacity . MPD is scaled using σ_con_ = 0.1 as reference and thus show the change factor of MPD at different σ_con_ values compared to the σ_con_ = 0.1 baseline. Model parameters that were kept constant for the simulations were: *K_0_*=10000; *σ_K_* =1; *µ*=0.01; σ_µ_ =0.02. To make the niche space comparable between the Gaussian and the quadratic case σ_K_ was set to 3 for the quadratic kernel. Figure produced in MATLAB version R2019a.

**Figure S6**

Mean γ-statistics as a function of niche widths (σα). Model parameters that were kept constant for the simulations were: K0=10000; σK =1; r = 1; µ=1e-3; σµ =0.02. Figure produced in MATLAB version R2019a.

**Figure S7**

Distribution of branch lengths (computed on 10 simulation replicates) as a function of niche width (σα). Model parameters that were kept constant for the simulations were: K0=10000; σK =1; r = 1; µ=1e-3; σµ =0.02. Figure produced in MATLAB version R2019a.

**Figure S8**

Distribution of species phylogenetic distances (computed on 10 simulation replicates) as a function of niche widths (σα). Model parameters that were kept constant for the simulations were: K0=10000; σK =1; r = 1; µ=1e-3; σµ =0.02.
